# Determining a Trustworthy Application for Medical Data Visualizations through a Knowledge-Based Fuzzy Expert System

**DOI:** 10.3390/diagnostics13111916

**Published:** 2023-05-30

**Authors:** Abdullah M. Albarrak

**Affiliations:** College of Computer and Information Sciences, Imam Mohammad Ibn Saud Islamic University, Riyadh 13318, Saudi Arabia; amalbarrak@imamu.edu.sa

**Keywords:** medical data analysis, trustworthy visualization tools, decision support systems, decision-making

## Abstract

Medical data, such as electronic health records, are a repository for a patient’s medical records for use in the diagnosis of different diseases. Using medical data for individual patient care raises a number of concerns, including trustworthiness in data management, privacy, and patient data security. The introduction of visual analytics, a computing system that integrates analytics approaches with interactive visualizations, can potentially deal with information overload concerns in medical data. The practice of assessing the trustworthiness of visual analytics tools or applications using factors that affect medical data analysis is known as trustworthiness evaluation for medical data. It has a variety of major issues, such as a lack of important evaluation of medical data, the need to process much of medical data for diagnosis, the need to make trustworthy relationships clear, and the expectation that it will be automated. Decision-making strategies have been utilized in this evaluation process to avoid these concerns and intelligently and automatically analyze the trustworthiness of the visual analytics tool. The literature study found no hybrid decision support system for visual analytics tool trustworthiness in medical data diagnosis. Thus, this research develops a hybrid decision support system to assess and improve the trustworthiness of medical data for visual analytics tools using fuzzy decision systems. This study examined the trustworthiness of decision systems using visual analytics tools for medical data for the diagnosis of diseases. The hybrid multi-criteria decision-making-based decision support model, based on the analytic hierarchy process and sorting preferences by similarity to ideal solutions in a fuzzy environment, was employed in this study. The results were compared to highly correlated accuracy tests. In conclusion, we highlight the benefits of our proposed study, which includes performing a comparison analysis on the recommended models and some existing models in order to demonstrate the applicability of an optimal decision in real-world environments. In addition, we present a graphical interpretation of the proposed endeavor in order to demonstrate the coherence and effectiveness of our methodology. This research will also help medical experts select, evaluate, and rank the best visual analytics tools for medical data.

## 1. Introduction

### 1.1. Overview

Per the study performed by Capminds, medical data privacy and visualization are two of the biggest challenges in the healthcare industry for the diagnosis of different crucial diseases [1]. The practice of analyzing current and historical data from the healthcare industry to forecast disease trends, increase outreach, and better manage the spread of illnesses is referred to as healthcare analytics [2]. Medical data visualization brings the most important takeaways in the health business into sharper focus, assists in the identification of patterns and connections for medical diagnostics, and makes data analysis more pertinent [3]. HIPAA (Health Insurance Portability and Accountability Act) specifies that there are almost 18 different aspects of PHI (protected health information) that need to be guaranteed. The most significant obstacle is removing these basic PHI components while maintaining the usefulness of the data for analysis. The HIPAA Security Rule requires organizations that store protected health information (PHI) to comply with a variety of specialized security measures. These measures include authentication methods, transmission security, restrictions on access, audits, and a variety of other procedures. [1]. The worldwide data management benchmark study for 2017 from Experian Data Quality states that, on average, C-level executives think that “33 percent of the medical data in their firms is erroneous [4].” Such kinds of erroneous or untrustworthy data can give the wrong diagnosis, thus harming the patient’s health.

**Figure 1:**
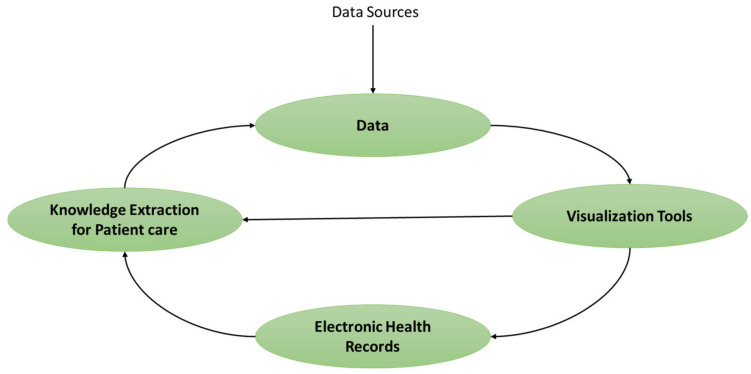
Integration of medical data and visualization tools.

Hence, it is not easy to gain trust in the data for a particular visualization. Additionally, “almost half of firms feel that a lack of trust in their medical data adds to an increased risk of non-compliance and regulatory penalties (52%), as well as a decline in patient loyalty (51%).” Of course, trust affects more than medical data corporations [5]. Visual analytics (VA) systems have shown substantial potential for solving information overload concerns in medical data, which they perform by integrating analytical approaches with interactive representations. Most healthcare organizations were unaware of the sources of the medical data they were using; according to a Bazaarvoice and Advertising Age poll on the topic, 75 percent of health experts lacked complete confidence in the ability of the medical data they were using to connect with their target audience, which is patients’ diagnoses [6]. Neither of these large businesses is the only one with this issue. The modern digital world, which includes tools, applications, mobile devices, smart home items, and smartwatches, has had a noteworthy impact on social existence. Further, the role of hybrid decision support systems in the visualization of medical data cannot be neglected. Figure 1 shows the tight integration of medical data, trustworthy visualization, and machine-learning-based decision support systems for knowledge discovery.

Decision support system-based smart visualization applications for medical data have given healthcare experts a sense of supremacy, greatly increasing their trustworthiness of other sources of data collection. These cutting-edge tools have changed not only how people live but also almost every other part of life. The fact that hybrid decision support systems are used in smart technologies today has led to the idea of trustworthy visualization tools as a way to keep medical data truthful [7,8,9,10]. Hybrid decision support systems give researchers and technical teams a set of methods comprising different algorithms that they can use to understand if the visual analytics tools they are using are consistent enough to obtain trustworthy medical data and diagnoses for patients [11,12,13,14]. Further, the selection and assessment of trustworthiness attributes with reference to visualization tools and hybrid decision support systems is a very difficult procedure based on various attributes, including data type and size, etc.

### 1.2. Background

This study looks at how trustworthiness is measured in visualization tools for medical data using the classifications described in the next sections. This research uses the unified fuzzy Analytic Hierarchy Process-Technique for Ordering Performance by Similarity to an Ideal Solution (AHP-TOPSIS), which is a well-known method for making decisions based on more than one factor [15,16,17,18,19,20,21]. Many authors and practitioners have used the AHP method to judge how well hybrid decision support systems work based on the value of one parameter or the value of another high-precision parameter. With the potential to speed up any problem analysis, AHP offers a practical method for establishing an initial linear approximation of this unexpressed utility function. Additionally, using the consistency metric to improve decision-maker learning has other advantages. Some of the benefits of TOPSIS approaches are their simplicity, logic, understandability, good processing efficiency, and ability to measure the relative performance of each choice in a simple mathematical way. However, combining the two for a problem-solving approach is difficult and takes much work.

According to Ghodsypour and O’Brien [9], AHP is a far superior method to other models for deciding how to prioritize analysis methods. The method might work because there is only a one-way hierarchy between the steps in making a decision. Carney and Wallnau [10] pointed out that it is more likely that the selection criteria for alternatives are related than that they are separate. An inaccurate outcome could be attained in such a challenging environment. Another useful method for resolving multiple-criteria decision-making (MCDM) problems is the TOPSIS [11] approach. The foundation of TOPSIS is the premise that the best option should also be closest to both the positive ideal solution (PIS) and the negative ideal solution (NIS). The assessment that goes along with the TOPSIS principle is simple and makes sense. Therefore, the inherent challenge of offering precise subjective assessments regarding the parameters must be acknowledged.

### 1.3. Contribution and Scope

This article discusses how the integration of hybrid decision support systems into trustworthy visual analytics tools for medical diagnosis has changed over time and what the key features are. It then uses the insights of experts in conversation regarding the pertinent key roles and responsibilities of trustworthiness, hybrid decision support systems, and visualization procedures that are quickly changing how healthcare industries perform patient care [9]. With the fuzzy AHP-TOPSIS method and a hybrid approach, the article will also give a scientific perspective on these roles and responsibilities [22,23,24,25,26,27]. The fuzzy AHP-TOPSIS method is a scientific MCDM method that has been used for a long time and has been proven to be accurate and useful [28,29,30,31,32,33,34,35].

- In order to accomplish the aforementioned objectives, this article suggests a novel method of trustworthiness evaluation for the visual analytics tools based on decision-making approaches for the correct diagnosis of diseases.
- We identified and examined the criteria that must be met in order to evaluate the trustworthiness of tools for medical data analysis in terms of visualization with hybrid decision support systems.
- We evaluated the effectiveness of the visualization characteristics of medical data and their trustworthiness through the fuzzy AHP method.
- To assess the overall impact of visual analytics tools, we used the fuzzy AHP TOPSIS method.
- To estimate the benefits and drawbacks of each visualization tool, we compared the results of the proposed method with those of other decision-making techniques.
- Based on the outcomes of the empirical investigation, we addressed unsolved problems and further recommended areas for future study in the data analysis of medical diagnostics.

### 1.4. Organization of the Paper

The complete structure of the research article is as follows. To summarize the subject and its importance, the Section 1 of this paper analyzes the numerous patterns and statistics from previous years. The Section 2 of the literature study discusses the commonly relevant studies conducted by earlier practitioners, which cover numerous characteristics in relation to the trustworthiness of visualization tools. In the Section 3, the authors go on to detail the many aspects of trustworthiness with respect to visualization tools, rank them in relation to previously established characteristics based on the possibility of impact, and provide a hierarchy. The authors conducted a numerical study of the proposed hierarchical problem using the fuzzy AHP-TOPSIS approach and analyzed the outcomes in the Section 4. The Section 5 summarizes the in-depth debate and lists the study’s shortcomings, with the Section 5 also coming to a conclusion.

## 2. Materials and Methods

### 2.1. Literature Review

A number of research papers on medical data visual analysis tool trustworthiness have explicitly used visualization and hybrid decision support systems; these combined approaches, which are meant to be innovative, have been mentioned in the proposed study. Throughout the previous ten years, researchers concentrated on the creation of various hybrid decision support systems for enhancing visualization tools’ trustworthiness. As the practice of decision systems based on expert opinions in visualization grows in trustworthiness, more and more researchers are adding hybrid decision support systems to their models of how medical data visual analytics works. A few established works from previous years are presented in Table 1 below.

Based on Table 1, it can be concluded that trustworthiness in visualizations of medical data is not a new issue to be noted. However, looking at the literature reveals that there has not been any significant work that addresses and presents a solution for the selection of the best attribute for the trustworthy visualization of medical data. Hence, trustworthiness in visualizations of medical data using hybrid decision support systems needs to be assessed for trust in medical data during human-machine interactions. Such multiple attribute issues can be solved using multiple criteria and decision-making algorithms.

Successful solutions to selection-based problems are achieved through fuzzy MCDM [19,20]. Since it can manage the information that is examined using a multi-resource linguistic and computational decision-making challenge and suggestions that are varied, the approach has been adopted by a number of researchers. Qianwen Wang et al. [21] conducted a survey of technologies. The authors discussed both the limitations of decision support systems and the limitations of this survey in their article. The explanation of machine learning constraints aids the study of decision support systems by providing academics and practitioners in visualization with a full introduction to decision support systems. The limitations of this study highlight the need for additional research in a number of exciting areas in order to further our understanding of decision support systems.

Samanlioglu et al. [22] combined fuzzy AHP, which uses Chang’s scale analysis [23], and fuzzy TOPSIS to provide a pathway for an IT company to choose the best employee applicant. In the analysis, intuitive fuzzy numbers were used to take into account the decision-makers’s (DM) stated thoughts. After using fuzzy AHP to figure out the values of different sub-attribute weights, they used fuzzy TOPSIS to compare five IT staff options based on fuzzy AHP weights.

Ciurana et al. [24] talked about their research on how to use a mixed fuzzy computational method to rate preservative manufacturing processes for micromanufacturing. Fuzzy TOPSIS was used to create ranks, while fuzzy AHP was used to create parameter weights. Nazam et al. [25] said that in a fuzzy environment, the TOPSIS procedure and a hybrid fuzzy AHP analysis could be used to rank and evaluate the risks of green supply chain management strategies. In the textile and auto industries, their hazy, risk-focused evaluation theory was put to the test. Lastly, the conceptual model reminds professionals and academics how important it is to perform thorough risk analyses before putting green supply chain interventions in place.

Xin Fu et al. [26] used a number of decision support systems to look at how well visualizations work. The authors integrated the visual analytics method with hybrid decision support systems. We employ a semi-supervised learning approach where decision support models are first trained for different assessment tasks, and then the variational autoencoder is used to extract valuable features from representations. Alicia Key et al. [27] introduced VizDeck, a web-based solution for visual analysis. Users can create context- and task-appropriate interactive visualization dashboards for their medical data with VizDeck. Our early usage of this mechanism with medical data from domain science research has also allowed users to quickly generate a large number of dashboards.

Several studies and tests have been performed to find out how well different decision support models work in different areas of the trustworthy visualization of medical data. Yet, the fuzzy AHP-TOPSIS method has not been used to look into how different decision support models for trustworthy visualizations of medical data systems affect the accuracy of medical data.

### 2.2. Integrating Hybrid Decision Support Systems into Trustworthy Visual Analytics Tools

Unquestionably, one of the most crucial components of medical data science is the visualization of medical data for explicit patient care. It is a crucial mechanism for examining and evaluating interactions among various parameters of trustworthiness and visualization [11]. Descriptive analytics can benefit from the visualization of medical data. Moreover, decision support systems employ an assessment of tools’ trustworthiness and visualization for feature selection, model testing, model construction, and model evaluation. It appears that decision support systems are currently very popular in the business world [13]. Almost every area of technology has undoubtedly been altered, and it has influenced daily life outside of the job. Decision support systems may be incorporated into the visualization mechanisms of medical data in a number of ways to help doctors and health experts enhance medical data analytics.

Since machine learning algorithms are made to automatically improve in analyzing medical data as they scan it, they are perfect for businesses that receive a steady stream of data [16]. Using the ranking of decision support systems for trustworthy visual analytics tools, analysts can see exactly what is happening at every stage of the trust chain and how threats or malicious events have an impact on medical data. With this level of detail, a visual analysis of medical data can help us learn more about consumer trust.

**Figure 2:**
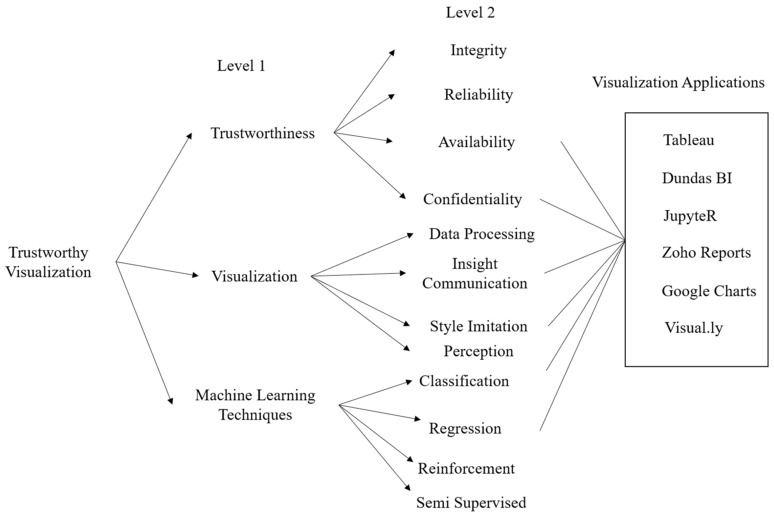
Hierarchy of characteristics for trustworthy visual analysis.

For authors to develop the best decision support systems, they must first organize the problem of medical data visual analytics tool trustworthiness into a hierarchy. There are different attributes that affect the user’s trust and the overall trustworthiness of the visualization of medical data. The decision-support systems that are to be used should also be composed around the problem. So, Figure 2 shows the problem as a hierarchy based on the trustworthy visualization of medical data, which consists of three levels of hierarchy.

Figure 2 represents the basic structure of trustworthy visualization, which is further divided into Level 1′s three attributes: trustworthiness, visualization, and machine learning techniques. The hierarchy is further divided into Level 2, which consists of the sub-attributes of trustworthiness, visualization, and machine learning techniques, respectively. For selecting the best alternative available according to the given attributes, we have taken six popular medical data visual analytics tools based on different machine learning algorithms, which are Tableau, Dundas BI, Jupyter, Zoho Reports, Google Charts, and Visual.ly [4]. The details of each attribute and sub-attribute from Level 1 and Level 2 are as follows.

The need for medical data sharing within and between businesses to facilitate analysis, mining, and decision-making is greater than ever before. Medical data must be trustworthy if health experts are to create correct analyses for explicit patient care and if decision-makers are to make effective decisions and take appropriate action. Trust may not always be an important aspect of interactions with the visualization of medical data, but it does become important when there is risk involved in using the information for a particular patient [8]. The visualization consists of two parts:

- Representation, which regards how information is turned from medical data into pictures and shown.
- Interaction, in which the user and the system have a conversation while the user examines the medical data set to find insights.

Both add to the end-user experience and are not mutually exclusive based on the idea of user intention. Further, the following are the three main key characteristics of trustworthy visualization:

- Trustworthiness: Trustworthiness of medical data refers to confidence in medical data, interpretation, and research quality procedures. The information is always linked to the trustworthiness assessment scores. A trust score is a numerical representation of trustworthiness that ranges from 0 to 1, with 0 denoting the least trustworthy and 1 denoting the most. Trustworthiness depends on four attributes: integrity, reliability, confidentiality, and availability [31]. There are multiple attributes of trustworthiness identified by different authors in the literature, but due to the complexity of medical data, we focused on the four most important. The details of these attributes are given in the next segment.
- Visualization: The technique of showing information and medical data in a graphic representation is referred to as visualization of medical data. Using maps, charts, and graphs, different types of visualization of medical data make it easy to spot trends, outliers, and patterns in medical data and determine what they mean. Additionally, it provides employees or business owners with a useful method for clearly conveying facts to non-technical audiences. In the world of big data, systems for the visualization of medical data are essential because they enable users to study enormous amounts of medical data and make data-driven decisions for patient care [5].
- Machine Learning Techniques: A type of artificial intelligence called “machine learning” tells computers how to learn from their own experiences. Machine learning algorithms use computational methods to “learn” information directly from medical data instead of using an equation as a model. As the number of examples available for learning grows, the algorithms automatically become more effective. A subset of machine learning is called deep learning [7]. There are different kinds of machine learning techniques that are derived from visualization, such as regression, classification, supervised, semi-supervised, reinforcement, dimension reduction, generative, etc. However, due to the complexity of the hierarchy, we have taken only four of the most commonly used techniques, which are regression, classification, semi-supervised learning, and reinforcement learning. Details of each learning technique are given in the following sections.

#### 2.2.1. Trustworthiness

The level of trustworthiness of medical data depends on how confident one is in the records, how they are interpreted, and the steps taken to ensure a diagnosis is good for patient care. Medical data are inextricably related to the results of the trustworthiness evaluation. Integrity, reliability, confidentiality, and availability are the four defining characteristics of trustworthiness [31]. The following section will go into further detail regarding these characteristics:

- Integrity: Medical data integrity is crucial because much depends on it. A single mistake in a medical data set can have far-reaching consequences for a patient’s health-wise decision-making processes. The accuracy, completeness, and consistency of medical data are referred to as data integrity. In terms of regulatory compliance, medical data integrity also relates to the security of medical data. Hence, it affects the overall trust of consumers, further affecting trustworthiness. There are a number of potential threats to the integrity of medical data. Medical data that have been copied or transferred should not be changed between updates. The integrity of medical data that are transferred or replicated without the intention of alteration is often ensured through error-checking methods and validation procedures.
- Reliability: Building trust in medical data across the enterprise requires complete and accurate medical data, which is the meaning of medical data reliability. One of the key goals of medical data integrity programs, which are also used to maintain medical data security, quality, and regulatory compliance, is to ensure medical data reliability. The term “medical data reliability” describes the degree to which information remains constant across different medical databases, applications, or platforms. It also relates to how reliable a medical data source is. A trustworthy statistic will always be accurate if the reliability of the medical data is high enough. Hence, it can be taken as an important attribute of trustworthiness.
- Confidentiality: The confidentiality of medical data implies shielding from prying eyes, both physically and digitally. Information privacy is related to confidentiality, including permission to access, distribute, and use it. Low-confidentiality information may be deemed “public” or otherwise harmless if shared with a wider audience. To avoid identity theft, account and system compromise, legal or reputational damage, and other serious consequences, information with high confidentiality concerns must be kept confidential. In this way, confidentiality is proven to be an important attribute in maintaining the trustworthiness of the visualization of data for medical diagnosis of critical diseases.
- Availability: Medical data availability is a gauge of how frequently medical data can be used, either internally or by a patient. Having access to medical data around the clock is ideal since it ensures the trustworthy diagnosis of any crucial disease. Medical data accessibility and the continuity of information supply are crucial elements of medical data availability. Inaccessible medical data are the same as absent or bad medical data. Hence, the trustworthiness of the visualization of medical data can be merely affected by the availability of medical data. A hospital and patient would suffer if their doctors and health experts had trouble accessing their medical data and finalizing a diagnosis.

#### 2.2.2. Visualization

Visualization is essential because it gives one the ability to learn, analyze, improve, standardize, and process medical data to obtain better results. Additional advantages of visualization are that it binds everyone collectively, communicates insights clearly, reduces complexity, records recommended practices, and brings more efficiency into the hospital for patient care. Further, the following are the main key procedures of the visualization process:

- Data Processing: Medical data analysis and visualization are two of the most important parts of making diagnostic and patient care decisions. Visualization-driven medical data processing helps healthcare experts who care about medical data find trends and make good treatment plans. Improved decision-making and observing changes in a doctor’s treatment behavior are just a few of the benefits of medical data analytics and visualization. Using medical data analytics and visualization approaches, businesses can examine various markets to determine which ones to target and which ones to ignore. Additionally, one of the most beneficial uses of visualization-driven medical data processing is in critical conditions and pandemic-like situations. Health experts can use it to analyze recent and historical trends to generate forecasts about the future direction of their treatment [32].
- Insight Communication: In the process of communicating insights, insights are turned into visual representations. We define “insight” as the understanding of the medical data that is conveyed through visual analysis, in line with other research in healthcare management systems [33]. The availability of insights is the primary distinction between insight communication and medical data display. In a medical data presentation, the underlying medical data contains insights that health experts must discover through visual investigation. On the other hand, in insight communication, insights are already available to designers and just need to be highlighted in the visualizations of medical data that are produced.
- Style Imitation: The practice of creating visualizations that are stylistically similar to samples provided is known as style imitation [9]. We refer to a group of factors as “style” to describe how they have an aesthetic impact on visualizations without having an impact on how the medical data are represented. A visualization’s style can be influenced by a variety of elements, including the color scheme, the decoration of the charts, and the design of the view.
- Visualization Perception: In visualization perception, users examine a visualization’s appearance, interpret the encoded medical data, and ascertain the content of the medical diagnosis [31]. Visualizations can now “read” similar to people on their own, thanks to machine learning-based decision support systems. This has aided designers in better understanding their health experts and allowed for the analysis of many representations.

#### 2.2.3. Machine Learning

One of the key areas of machine learning is understanding medical data and visualizing them accordingly for the end user. In order to solve real-world problems, we must take much raw medical data and turn them into relevant knowledge. With the aid of visualization of medical data, we can see how the records appear and what type of association the qualities of the records hold, transforming raw medical data into relevant knowledge for a patient or forecast diseases. We can comprehend machine-learning medical data with statistics by using machine-learning recipes as a guide. The following are the four main key learning techniques of machine learning:

- Classification: Recognition, comprehension, and grouping of items and concepts into predetermined groupings, or “sub-populations,” is the process of classification [24]. Machine learning systems use a variety of methods to put new data sets into categories using these training data sets that have already been put into categories. Based on training data, classification algorithms in machine learning can guess how likely it is that the next piece of data will fit into one of the categories. One of the most common uses of categorization is to sort emails into “spam” or “non-spam” folders, which is what the best email service providers provide today.
- Regression: Regression analysis describes the relationship between independent (predictor) and dependent (target) variables using one or more independent variables [25]. While all other independent variables remain constant, regression analysis enables us to understand how the value of a dependent variable varies in relation to an independent variable. Variables that are constant or real, such as temperature, age, pay, and cost, are included in predictions.
- Reinforcement: Reinforcement learning teaches computers to perform what you ask of them by rewarding good behavior and/or punishing bad behavior [18]. The standard capabilities of a reinforcement learning agent include the ability to observe and comprehend its environment, the ability to act, and the ability to learn from mistakes. Reinforcement learning formalizes this process of teaching “agents” to maximize a “reward.” This “reward” is produced using a function that rewards actions we want to encourage and punishes those we want to discourage. In contrast to other machine learning paradigms where the loss function is a powerful signal to direct model behavior, the reward function in the reinforcement learning scenario is, at most, a proxy signal to direct the agent toward optimal behavior. In many ways, the statement in this form makes the problem more difficult to solve.
- Semi-supervised Learning: This is a practice for learning from both a large number of unlabeled examples and a small number of examples that have been labeled [15]. These kinds of learning problems are hard because neither supervised nor unsupervised learning algorithms can use both labeled and unlabeled data in a good way. This necessitates the use of specific, semi-supervised learning methods. The area of machine learning, known as semi-supervised learning, is focused on performing specific learning tasks utilizing both labeled and unlabeled data.

### 2.3. Proposed Methodology

This section describes a method for choosing trustworthy visual analytics tools for medical data analysis. Three steps make up the strategy [33,34]. The paper must first establish a thorough hierarchy of all the variables that could affect the problem. This is performed by thoroughly inspecting the chain in question and identifying any possible faults. The results are then grouped using similar traits after the overlaps have been determined. Anytime there is a significant change to the chain, this exercise should be performed. The next step in the procedure is to assign weights to the criteria based on how relevant they are. Fuzzy AHP is used for this, and professional opinions are taken into account. Examining the results of several criteria under specific key categories is part of the third phase. The fourth step, which determines if the organization is prepared to use raw materials, uses the fuzzy TOPSIS technique.

#### 2.3.1. Fuzzy AHP

The fuzzy AHP practice expands Saaty’s idea of AHP by combining it with fuzzy set theory [34,35]. In fuzzy AHP, fuzzy scales are used to illustrate the comparative intensity of the various criteria’s contributing components. A fuzzy decision matrix can be created as a result. Fuzzy quantities are also used to embody the final scores of alternatives [34]. Applying particular algebraic operators to the fuzzy integers yields the optimal solution. In this way, these fuzzy integers show all of the parts of the weight vectors and the judgment matrix.

**Figure 3:**
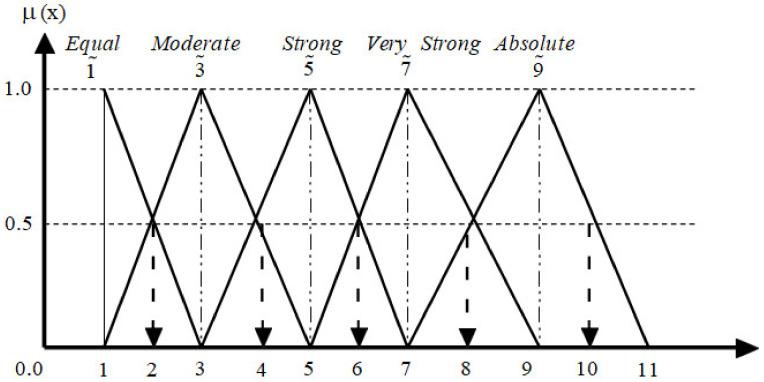
Function of fuzzy membership.

The relative relevance of one kind compared to another is then reflected in a fuzzy judgment matrix for each measure using fuzzy numbers [31]. Using these evaluation vectors and the fuzzy pair-wise comparison matrix, we may assign relative importance to each criterion. Table 2 shows linguistic utterances represented as fuzzy integers, and Table 3 shows the consistency ratio (CR) computed using the random index (RI). For both the criteria and the sub-criteria, the fuzzy membership function for linguistic terms is depicted in Figure 3. The final weights are arrived at once the linguistic term arrangement has been arranged, altered, and reviewed by experts.

#### 2.3.2. Fuzzy TOPSIS

Applying fuzzy AHP and fuzzy TOPSIS entails the following three procedures: one must first discover what elements are having an impact on the DM procedure, then perform the calculations for the fuzzy AHP, and finally utilize the fuzzy TOPSIS and Table 4 to rank the options.

In the first phase, we identify potential characteristics for the underlying system, identify criteria that would have an impact, and establish a hierarchy for making decisions. When this phase is complete, the decision-makers team will have approved the DM chain of command. One of the most important contributions of the proposed strategy is its capacity to deal with ambiguity in regard to criteria in general and resources in particular. The following characteristics are used in the method to decrease or remove uncertainty:

- Ensuring that the variables in each part of the model are the same so one can determine how well the experts in the field can solve the problem.
- Replacing hard numbers with words by applying two different fuzzy membership functions to the problems we have talked about
- Taking into account more than one weighted source that is related to the topic at hand, which makes the medical data less uncertain.
- Using its two-module structure to handle different levels of granularity and uncertainty in medical data sources, such as exact qualitative and intangible quantitative medical data, as well as tangible quantitative medical data and data from field surveys.

The fuzzy TOPSIS technique is well suited to dealing with real-world application issues in a fuzzy environment. One of the traditional multi-attribute-based DM systems, TOPSIS [33], was created. It is based on the premise that the alternative that is picked should be the one that is the furthest from both the PIS and NIS solutions. Additionally, TOPSIS features a flexible and simple-to-use calculation method. It has the capacity to take into account many criteria with different units at once [32]. The steps of the fuzzy AHP-TOPSIS estimate are computed below.

Step 1: Determine the relative importance of each criterion for evaluation. In this study, the fuzzy AHP is used to determine preference weights.

Step 2: Build the fuzzy decision/performance matrix and use the criteria to select the right linguistic variables for the different options (Equations (1) and (2)).

(1) $$\tilde{DA}=\left[\begin{array}{c} F_{1}\\ F_{2}\\ \vdots \\ F_{m} \end{array}\right]\left[\begin{array}{ccccc} f_{11} & f_{12} & \ldots & \ldots & f_{1n}\\ f_{21} & f_{22} & \ldots & \ldots & f_{2n}\\ \ldots & \ldots & \ldots & \ldots & \ldots \\ \ldots & \ldots & \ldots & \ldots & \ldots \\ f_{m1} & f_{m2} & & & f_{mn} \end{array}\right]$$

where *i* = 1, 2, 3, …*m* and *j* = 1, 2, …*n*.

(2) $$x_{ij}=\frac{1}{K}(x_{ij}^{-1}⊕\ldots ⊕x_{ij}^{-k}⊕\ldots x_{ij}^{-K})$$

where $x_{ij}^{-k}\;$ is the performance rating of attribute $A_{i}$ with respect to $C_{j}$ evaluated by the kth expert and ${\tilde{x}}_{ij}^{k}=(l_{ij}^{k},m_{ij}^{k},u_{ij}^{k})$.

Step 3: Construct the fuzzy decision matrix. The unprocessed medical data are normalized by employing a linear scale conversion in order to bring the scales of the different criteria into a comparable format. The fuzzy decision matrix for the attributes $\left(\tilde{DA}\right)$ is built as follows (Equations (3)–(5)):

(3) $$\left(\tilde{DA}\right)={\left[{\tilde{d}}_{ij}\right]}_{m\times n}$$

where *i* = 1, 2, 3, …*m* and *j* = 1, 2, …*n*.

(4) $${\tilde{d}}_{ij}=\left(\frac{x_{ij}}{z_{j}^{*}},\frac{y_{ij}}{z_{j}^{*}},\frac{z_{ij}}{z_{j}^{*}}\right)\;\mathrm{and}\;z_{j}^{*}=\underset{i}{\max}z_{ij}$$

(5) $${\tilde{d}}_{ij}=\left(\frac{x_{j}^{-}}{z_{ij}},\frac{x_{j}^{-}}{y_{ij}},\frac{x_{j}^{-}}{x_{ij}}\right)\;\mathrm{and}\;z_{j}^{*}=\underset{i}{\max}z_{ij}$$

Step 4: Build the weighted and normalized matrix.

Multiplying the weights (wj) of calculating criteria by the normalized fuzzy decision matrix is how one arrives at the weighted normalized matrix (wj) for criteria. ${\tilde{d}}_{ij}$ (Equation (6)).

(6) $$\tilde{V}={\left[v_{ij}\right]}_{m\times n}\;i=1,\;2,\;\ldots n;\;j=1,\;2,\;3\;\ldots m$$

where $v_{ij}={\tilde{d}}_{ij}\left(\cdot \right)W_{j}$.

Note that ${\tilde{v}}_{ij}$ is a TFN represented by $\left({\tilde{x}}_{ijk},{\tilde{y}}_{ijk},{\tilde{z}}_{ijk}\right)$.

Step 5: Calculation that is used to determine the fuzzy PIS and fuzzy NIS of the attributes (Equations (7) and (8)):

(7) $$F^{*}=\left({\tilde{v}}_{1}^{*},{\tilde{v}}_{2}^{*}\ldots \ldots .{\tilde{v}}_{n}^{*}\right)$$

where ${\tilde{v}}_{j}^{*}=\left({\tilde{z}}_{j}^{*},{\tilde{z}}_{j}^{*},{\tilde{z}}_{j}^{*}\right)\;{\tilde{z}}_{j}^{*}=\underset{i}{\max}\left\{{\tilde{z}}_{ij}\right\}$

(8) $$F^{-}=\left({\tilde{v}}_{1}^{-},{\tilde{v}}_{2}^{-}\ldots \ldots .{\tilde{v}}_{n}^{-}\right)$$

where ${\tilde{v}}_{j}^{-}=({\tilde{x}}_{j}^{-},{\tilde{x}}_{j}^{-},{\tilde{x}}_{j}^{-})\;{\tilde{x}}_{j}^{-}=\underset{i}{\min}\left\{{\tilde{x}}_{ij}\right\}$; i = 1, 2, 3 …m; j = 1, 2, …n.

Step 6: Determine the distance between individual attributes.

The distance ($d_{i}^{+}$,$\;d_{i}^{-}$) of individual weighted alternative *i* = 1, 2, 3 …*m* from the fuzzy PIS and fuzzy NIS is computed as per the following (Equations (9)–(11)):

(9) $$d_{i}^{+}=\sum_{j=1}^{n}dv\left({\tilde{v}}_{ij},{\tilde{v}}_{j}^{+}\right)\;i=1,\ldots \;\ldots ,m$$

(10) $$d_{i}^{-}=\overset{n}{\underset{j=1}{\sum }}dv\left({\tilde{v}}_{ij},{\tilde{v}}_{j}^{-}\right)\;i=1,\ldots \;\ldots ,m$$

(11) $$d(\tilde{A},\tilde{B})=\sqrt{\frac{1}{3}({\left(x_{A}-x_{B}\right)}^{2}+{\left(y_{A}-y_{B}\right)}^{2}+{\left(z_{A}-z_{B}\right)}^{2})}$$

Step 7: Computation of the closeness coefficient $ClCo_{i}$ of the individual alternative.

The closeness coefficient $ClCo_{i}$ symbolizes the distances to the fuzzy PIS ($d^{+}$) and the fuzzy NIS ($d^{-}$) concurrently. The closeness coefficient of separate attributes is measured as (Equation (12)):

(12) $$ClCo_{i}=\frac{d_{i}^{-}}{\left(d_{i}^{+}+d_{i}^{-}\right)}$$

Step 8: Rank the attributes.

In step 8, the values of the maximum closeness coefficient are used to rank or choose the different attributes in decreasing order.

## 3. Results

This section discusses the different measurements of the AHP-TOPSIS method’s integrated fuzzy deployment. In our research study, we employed the integrated fuzzy AHP-TOPSIS approach, which is a well-known and proven method for making decisions. This method is meant to compare the effectiveness of different trustworthy visual analytics tools based on how they work in the modern world of data analysis for correct and trustworthy medical diagnoses. To come up with more validating results, we asked 151 experts, with the help of questionnaires, in different fields and technologies for their advice. These experts belong to the fields of medical data, professional healthcare, and healthcare security expertise. According to Figure 2, at Level 1, three parameters were set up for trustworthy visualization: trustworthiness (C1), visualization (C2), and machine learning techniques (C3). These were used to compare the different alternatives for trustworthy visualization. Further sub-parameters for trustworthiness were integrity (C11), reliability (C12), availability (C13), and confidentiality (C14). The visualization parameters were data processing (C21), insight communication (C22), style imitation (C23), and perception (C24). Sub-parameters for the machine learning techniques were classification (C31), regression (C32), reinforcement (C33), and semi-supervised (C34). Six alternatives to effective visual analytics tools for medical data were Tableau, Dundas BI, Jupyter, Zoho Reports, Google Charts, and Visual.ly. Further, pair-wise comparison matrices were used to generate the weights for the local criterion and sub-criteria. With the help of Section 4, the impact assessment of trustworthy visualization by utilizing AHP-TOPSIS in a fuzzy environment was investigated as follows.

The authors of this study first turned the expert opinions from linguistic values into numbers, and then, with the help of Section 4, Table 2, and Table 3 as guides, they turned the numbers into fuzzy-based crisp numbers. The final findings were then calculated numerically to produce the pair-wise comparison matrix, and they are shown in Table 5. Equation (1) was used to set up fuzzy wrappers; Equation (2) was used to figure out the triangular numbers; and Equation (3) was used to figure out the first and second type weights, which have values between 0 and 1. The experts then calculated the pair-wise comparison matrix. As indicated in Table 5, the pair-wise comparative matrix for the Level 1 hierarchy was developed. Additionally provided are the various pair-wise relative matrices for the Level 2 hierarchical diagram in Table 5. The steps from Section 4 were used to figure out the defuzzified values and normalized weights of the Level 1 attributes, and the results are shown in Table 2. To properly understand how to calculate Table 2, the following steps must be taken. The pair-wise comparison matrices were initially combined into defuzzified values. Defuzzified values were examined and transformed into group weights. Table 6 shows the defuzzification matrix and normalized weights. Additionally, Level 2 aggregated pair-wise comparison matrices are shown in Table 6. In order to get the final weights of each attribute or characteristic, integration was especially used. Table 6 uses a hierarchical framework to display the overall weights and ranks of the characteristics with respect to levels.

**Figure 4:**
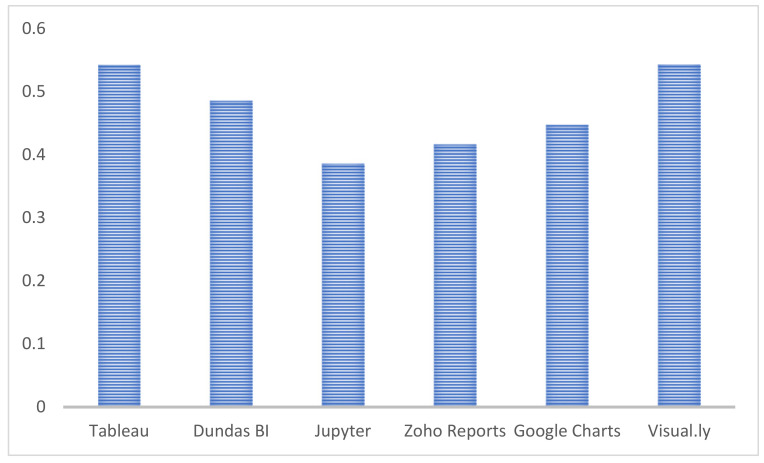
Impact of alternatives.

The fuzzy-based AHP technique was used to find the defuzzified and normalized weights of each characteristic, and the fuzzy TOPSIS method was used to make a global ranking of competing options. We collected the technological inputs for six visual analytics tools (Tableau 2019.2 version, Dundas BI, Jupyter, Zoho Reports, Google Charts, and Visual.ly) using the standard scale in Table 4 and Equations (1)–(3) from the Section 2.3. In a fuzzy environment, the weights of attributes found by the fuzzy method and AHP were specified for the TOPSIS technique to find a different ranking. In Table 7, a normalized fuzzy decision matrix for six different and competitive alternatives is generated with the aid of Equations (4)–(7) and provided. The normalized fuzzy decision-matrix cell values (performance values) were multiplied by each attribute weight value using Equations (8) and (9), and a weighted fuzzy normalized decision-matrix was created, as shown in Table 8. The calculations are displayed in Table 9 and Table 10 after applying Equations (10) and (11) to determine the positive and negative idealness of each alternative with regard to each attribute. The final results are displayed in Table 11 and Figure 4 after using Equation (12) and computing the relative closeness score for each alternative. As a result, Visual.ly, which has an efficiency score of 0.5436 among the various alternatives, was determined to be the most effective among the six compared options.

The ranking of competitive alternatives was generated through TOPSIS of alternatives, Tableau, Dundas BI, Jupyter, Zoho Reports, Google Charts, and Visual.ly, based on the preference score or relative closeness score. According to the results of this analysis, Visual.ly offers a better application to address key problems of trustworthiness of medical diagnosis and challenges based on predetermined criteria than other applications. Further, it was demonstrated by fuzzy decision-making in estimating trustworthy visualization.

## 4. Discussion

All modern medical institutes must have data visualization tools for good patient care. Healthcare data analytics will grow 3.5 times, from $11.5 billion in 2019 to $40.8 billion in 2025 [32]. Medical data, such as electronic health records (EHR), is massive and will only grow. Large amounts of medical data have become vital to varied groups of experts in domains including economics, space exploration, and climate change, as well as to individuals and communities. Medical data provides vital information. The visualization technology of data lets clinicians create a 3D image of MRI scans to get clearer pictures of blood vessels, organ tissues, and bones without surgery. Savings in time and money are significant.

Our study suggested a new way to determine how visual tools for medical data can help doctors help patients. After looking at different visualization tools, we found that the Visual.ly visualization tool, which meets the needs of both patients and healthcare professionals, is a reliable tool. A comparison of different MCDM methods was also performed to verify the results of this study.

Using a wide range of different analysis methods can give a clear answer to the question of whether the analyzed result and the planned method are better or not. The author of this study has compared the results of the fuzzy AHP-VIekriterijumsko KOmpro-misno Rangiranje (VIKOR) and fuzzy AHP-Elimination Et Choix Traduisant la Realité (ELECTRE) methodologies [2,6,9] with the results of the fuzzy AHP-TOPSIS technique to gauge the effectivity of the proposed methodology. The results from fuzzy AHP-VIKOR and fuzzy AHP-ELECTRE are similar to the results from fuzzy AHP-TOPSIS. Table 12, which one can see below, shows the differences between the two groups.

The authors conducted a comparison with other approaches (fuzzy AHP-VIKOR and fuzzy AHP ELECTRE) already in use; in the comparison, the authors used the same medical data to assess the other methods accessible in the study [12]. In Table 12, comparison findings with other approaches are shown, and it is clear from the medical data that the fuzzy AHP-TOPSIS method performs better than other methods used in the study.

Based on Table 12, fuzzy AHP TOPSIS yields more useful results than any other approach. Thus, the fuzzy AHP-TOPSIS hybrid technique is better for solving this problem. Several studies have examined the reliability of medical data visualization technologies. The visualization tools’ dependability for medical data in fuzzy scenarios has not been quantified.

## 5. Conclusions

Medical data analysis trustworthiness issues prevent data summaries of specific patients from different healthcare provider databases. Medical data analysis makes sharing medical data between healthcare providers easy [20,21]. Since medical databases are linked directly to a healthcare provider or regional boundaries, it is difficult to locate patient records from other medical data. They use centralized storage, which is not that trustworthy for any kind of disease diagnosis. Selecting an effective, trustworthy visualization tool for medical data diagnosis is crucial. Given that there are so many visual analytics tools available, choosing a trustworthy one among them might be difficult because each tool has its own benefits and drawbacks.

The findings of a study on the use of the fuzzy AHP-TOPSIS methodology were put into practice. Based on the literature review, a set of criteria was discovered and arranged into a logical hierarchical structure with twelve primary criteria and six alternatives. For all experts, the consistency ratios in the gathered reply forms were less than 0.10. According to the study’s findings, when it comes to choosing a trustworthy tool, Visual.ly (with a closeness coefficient value of 0.5436) outperforms Tableau (0.5425), Dundas BI (0.4856), Jupyter (0.3861), Zoho reports (0.4163), and Google charts (0.4474). Choosing the appropriate weights for the crucial motive, purpose, and consciousness emphasis areas can improve the creation of trustworthy visualizations for medical data and aid in decision-making and policy compliance. The fuzzy AHP-TOPSIS results that are reported in this work can be utilized to choose or create efficient diagnoses for critical patients using visual analysis tools that could help health experts. Further limitations of the research work are as follows:

- Other trustworthiness, visualization, and machine learning factors may also have an impact on these weights.
- Other fuzzy decision-making methods, such as fuzzy PROMETHE-I, fuzzy PROMETHE-II, and many others, are also available. Their results can be compared to those of this study by using these methods in future research.

Decision-making methods such as fuzzy AHP TOPSIS have shown promise for ranking visualization tools for medical data, but there is still much room for exploration. The same approach can be used in other areas of health research in the future. The study can be expanded to evaluate the effectiveness of the methods on more datasets, especially larger ones. To better address the challenges of data computation time and reap the benefits of big data, more research should focus on this area. Researchers in the future may use a variety of approaches that use different machine learning algorithms to fine-tune the proposed model in light of fresh data.

## Figures and Tables

**Table 1:** Pertinent works of visual analytics tools.

Authors	Year	Visual Analytics Tools	Results
Daniel Keim et al. [13]	2008	Semantics-based approaches	Raised issues with the reliability and adequacy of medical data in visual analytics.
A. Endert et al. [14]	2018	Various decision support systems	Proposed sensemaking loop for visualization of medical data through various decision support systems
Dominik Sacha et al. [15]	2016	Review of various decision support systems for trustworthy visualizations of medical data	Dimensionality reduction methods for trustworthy visualizations of medical data are great exploratory methods.
Dominik Sacha et al. [16]	2014	Hybrid decision support systems	Proposed a detailed knowledge generation model that integrates humans and systems for different decision support systems in trustworthy visualizations of medical data
Mennatallah El-Assady et al. [17]	2019	Optimization algorithms	Presented a visual analytics framework for mixed-initiative speculative execution-based topic model optimization.
Felix Grüun et al. [18]	2016	Convolutional Neural Networks	Proposed an easy-to-use open-source library named FeatureVis library for MatConvNet.
Emma Beauxis-Aussalet et al. [20]	2021	Various decision support systems	Claimed that trustworthy visualizations of medical data cannot address all aspects of trust.
R Boadh et al. [36]	2022	Different fuzzy expert systems	Reviewed various fuzzy experts systems for the diagnosis of critical diseases

**Table 2:** Scale.

Numeric Value	Verbal Value	Triangular Fuzzy Number (TFN)
1	Equally significant	1, 1, 3
3	Less significant	1, 3, 5
5	Very significant	3, 5, 7
7	Incredibly Essential	5, 7, 9
9	Extremely Essential	7, 9, 11

**Table 3:** Scale of random index.

Size (n)	1	2	3	4	5	6	7	8
RI	0	0	0.52	0.89	1.11	1.25	1.35	1.40

**Table 4:** Rating scale.

Verbal Values	Equivalent TFNs
Very Poor (VP)	1, 1, 3
Poor (P)	1, 3, 5
Medium (M)	3, 5, 7
Good (G)	5, 7, 9
Very Good (VG)	7, 9, 11

**Table 5:** Pair-wise comparisons matrixes of the groups.

Characteristic A/Characteristic B	Fuzzy Pair-Wise Comparisons Matrixes	Defuzzified Pair-Wise Comparisons Matrixes
Trustworthiness/Visualization	0.9710, 1.2475, 1.6094	1.2689
Trustworthiness/Machine Learning Techniques	1.0592, 1.5849, 2.2206	1.3124
Visualization/Machine Learning Techniques	0.6352, 0.9143, 1.3430	0.9693
Integrity/Reliability	0.8206, 1.1118, 1.6150	1.1648
Integrity/Availability	0.3230, 0.4480, 0.6051	0.4561
Integrity/Confidentiality	0.5670, 0.7132, 0.8739	0.7168
Reliability/Availability	0.6600, 1.1700, 1.6900	1.1730
Reliability/Confidentiality	1.1500, 1.4400, 1.7000	0.4940
Availability/Confidentiality	0.4900, 0.6400, 1.0000	1.1720
Data Processing/Insight Communication	1.0000, 1.5200, 1.9300	0.8920
Data Processing/Style Imitation	0.2200, 0.2900, 0.4200	0.6910
Data Processing/Perception	0.6900, 0.8900, 1.1000	0.3720
Insight Communication/Style Imitation	0.3000, 0.4400, 0.8000	0.8533
Insight Communication/Perception	0.2300, 0.2800, 0.3600	0.7337
Style Imitation/Perception	0.4900, 0.6400, 1.0000	0.6707
Classification/Regression	0.8206, 1.1118, 1.6150	1.1648
Classification/Reinforcement	0.2200, 0.2900, 0.4200	0.6910
Classification/Semi-Supervised	0.6900, 0.8900, 1.1000	0.3720
Regression/Reinforcement	0.3000, 0.4400, 0.8000	0.4561
Regression/Semi-Supervised	0.2300, 0.2800, 0.3600	0.7168
Reinforcement/Semi-Supervised	0.4900, 0.6400, 1.0000	1.1730

**Table 6:** Weights of Trustworthy Visualization.

Characteristic	Symbols	Independent Weight of the Groups	Overall Weights through Hierarchy	Percentage	Priority
Characteristics of Group 1 at Level 1					
Trustworthiness	C1	0.392181	0.392181	39.22%	1
Visualization	C2	0.302458	0.302458	30.25%	3
Machine Learning Techniques	C3	0.305361	0.305361	30.54%	2
Characteristics of Groups 1, 2, and 3 at Level 2					
Integrity	C11	0.189388	0.074274	7.43%	8
Reliability	C12	0.210311	0.082480	8.25%	6
Availability	C13	0.300182	0.117726	11.77%	1
Confidentiality	C14	0.300120	0.117701	11.77%	2
Data Processing	C21	0.166168	0.050259	5.03%	12
Insight Communication	C22	0.220006	0.066543	6.65%	9
Style Imitation	C23	0.246062	0.074423	7.44%	7
Perception	C24	0.367763	0.111233	11.12%	3
Classification	C31	0.177183	0.054105	5.41%	10
Regression	C32	0.172464	0.052664	5.27%	11
Reinforcement	C33	0.327841	0.100110	10.01%	4
Semi-Supervised	C34	0.322513	0.098483	9.85%	5

**Table 7:** Normalized decision matrix for alternatives with respect to criteria.

	Tableau	Dundas BI	Jupyter	Zoho Reports	Google Charts	Visual.ly
Integrity	0.6578	0.8500	0.6578	0.7720	0.1000	0.6490
	0.7570	0.9170	0.7570	0.8560	0.1920	0.7640
	0.9190	0.9680	0.9190	0.9450	0.3840	0.8800
Reliability	0.6570	0.6570	0.6578	0.8500	0.6578	0.5740
	0.7650	0.7650	0.7570	0.9170	0.7570	0.7220
	0.9050	0.9050	0.9190	0.9680	0.9190	0.0820
Availability	0.6490	0.6578	0.6570	0.4560	0.8500	0.7720
	0.7640	0.7570	0.7650	0.5330	0.9170	0.8560
	0.8800	0.9190	0.9050	0.7330	0.9680	0.9450
Confidentiality	0.5740	0.6490	0.6570	0.6578	0.8500	0.6578
	0.7220	0.7640	0.7650	0.7570	0.9170	0.7570
	0.0820	0.8800	0.9050	0.9190	0.9680	0.9190
Data Processing	0.1000	0.6490	0.6578	0.6570	0.4560	0.8500
	0.1920	0.7640	0.7570	0.7650	0.5330	0.9170
	0.3840	0.8800	0.9190	0.9050	0.7330	0.9680
Insight Communication	0.6578	0.8500	0.6490	0.6570	0.4560	0.5740
	0.7570	0.9170	0.7640	0.7650	0.5330	0.7220
	0.9190	0.9680	0.8800	0.9050	0.7330	0.0820
Style Imitation	0.6570	0.6570	0.6490	0.6578	0.6570	0.4560
	0.7650	0.7650	0.7640	0.7570	0.7650	0.5330
	0.9050	0.9050	0.8800	0.9190	0.9050	0.7330
Perception	0.6578	0.6578	0.8500	0.6490	0.6570	0.4560
	0.7570	0.7570	0.9170	0.7640	0.7650	0.5330
	0.9190	0.9190	0.9680	0.8800	0.9050	0.7330
Classification	0.6490	0.6578	0.6570	0.4560	0.8500	0.6578
	0.7640	0.7570	0.7650	0.5330	0.9170	0.7570
	0.8800	0.9190	0.9050	0.7330	0.9680	0.9190
Regression	0.8500	0.6490	0.6490	0.6578	0.6570	0.4560
	0.9170	0.7640	0.7640	0.7570	0.7650	0.5330
	0.9680	0.8800	0.8800	0.9190	0.9050	0.7330
Reinforcement	0.6570	0.6578	0.8500	0.6490	0.6570	0.4560
	0.7650	0.7570	0.9170	0.7640	0.7650	0.5330
	0.9050	0.9190	0.9680	0.8800	0.9050	0.7330
Semi-Supervised	0.6578	0.6570	0.6570	0.6578	0.8500	0.6578
	0.7570	0.7650	0.7650	0.7570	0.9170	0.7570
	0.9190	0.9050	0.9050	0.9190	0.9680	0.9190

**Table 8:** Weighted normalized decision matrix.

	Tableau	Dundas BI	Jupyter	Zoho Reports	Google Charts	Visual.ly
Integrity	0.0230	0.0630	0.0630	0.0610	0.0230	0.1330
	0.0430	0.1140	0.0979	0.0870	0.0430	0.1840
	0.0550	0.1310	0.1310	0.1200	0.0550	0.2080
Reliability	0.0580	0.0230	0.0230	0.0630	0.0516	0.0580
	0.0850	0.0370	0.0370	0.0979	0.0820	0.0850
	0.0950	0.0430	0.0430	0.1140	0.0990	0.0950
Availability	0.0230	0.0630	0.0516	0.0580	0.0630	0.0610
	0.0370	0.0979	0.0820	0.0850	0.0979	0.0870
	0.0430	0.1140	0.0990	0.0950	0.1310	0.1200
Confidentiality	0.0230	0.0630	0.0230	0.0630	0.0516	0.0580
	0.0370	0.0979	0.0370	0.0979	0.0820	0.0850
	0.0430	0.1140	0.0430	0.1140	0.0990	0.0950
Data Processing	0.0890	0.1330	0.0890	0.1330	0.0580	0.0630
	0.0960	0.1680	0.0960	0.1680	0.0850	0.0979
	0.1030	0.2080	0.1030	0.2080	0.0950	0.1310
Insight Communication	0.0630	0.0516	0.0630	0.0516	0.0630	0.0516
	0.1140	0.0990	0.1140	0.0990	0.0979	0.0820
	0.1310	0.1220	0.1310	0.1220	0.1140	0.0990
Style Imitation	0.0230	0.0230	0.0630	0.0230	0.0630	0.0516
	0.0370	0.0370	0.0979	0.0370	0.0979	0.0820
	0.0550	0.0430	0.1140	0.0430	0.1140	0.0990
Perception	0.1330	0.0890	0.1330	0.0890	0.1330	0.0580
	0.1680	0.0960	0.1680	0.0960	0.1680	0.0850
	0.2080	0.1030	0.2080	0.1030	0.2080	0.0950
Classification	0.0230	0.0630	0.0516	0.0630	0.0516	0.0630
	0.0370	0.1140	0.0990	0.1140	0.0990	0.0979
	0.0430	0.1310	0.1220	0.1310	0.1220	0.1140
Regression	0.0890	0.0230	0.0230	0.0230	0.0630	0.0516
	0.0960	0.0370	0.0370	0.0370	0.0979	0.0820
	0.1030	0.0550	0.0430	0.0430	0.1140	0.0990
Reinforcement	0.0610	0.1330	0.0890	0.1330	0.0630	0.0230
	0.0870	0.1680	0.0960	0.1680	0.0979	0.0370
	0.1200	0.2080	0.1030	0.2080	0.1140	0.0430
Semi-Supervised	0.0580	0.0516	0.0630	0.0516	0.1330	0.0890
	0.0850	0.0990	0.1140	0.0990	0.1680	0.0960
	0.0950	0.1220	0.1310	0.1220	0.2080	0.1030

**Table 9:** Separation from positive solution.

	Tableau	Dundas BI	Jupyter	Zoho Reports	Google Charts	Visual.ly
Integrity	0.9280	0.9360	0.9280	0.9360	0.9080	0.9280
Reliability	0.9280	0.9360	0.9080	0.9280	0.9050	0.8990
Availability	0.9280	0.9360	0.9080	0.9280	0.9080	0.9280
Confidentiality	0.9360	0.9080	0.9280	0.9360	0.9080	0.9280
Data Processing	0.9360	0.9080	0.9280	0.9360	0.9080	0.9280
Insight Communication	0.9080	0.9050	0.8990	0.9080	0.9050	0.8990
Style Imitation	0.9360	0.9360	0.9360	0.9080	0.9280	0.9080
Perception	0.9080	0.9280	0.9080	0.9280	0.9360	0.9080
Classification	0.9050	0.9280	0.9080	0.9360	0.9080	0.9280
Regression	0.9050	0.9360	0.9080	0.9080	0.9280	0.9360
Reinforcement	0.9080	0.9360	0.9360	0.9080	0.9280	0.9080
Semi-Supervised	0.9050	0.9080	0.9080	0.9280	0.9360	0.9080

**Table 10:** Separation from negative solution.

	Tableau	Dundas BI	Jupyter	Zoho Reports	Google Charts	Visual.ly
Integrity	0.1420	0.0012	0.0150	0.1730	0.0150	0.0190
Reliability	0.0120	0.0140	0.1420	0.0012	0.1420	0.0140
Availability	0.2410	0.1230	0.1420	0.0012	0.0450	0.0150
Confidentiality	0.1420	0.0012	0.0150	0.1420	0.0012	0.0150
Data Processing	0.0120	0.0140	0.1420	0.0120	0.0140	0.1420
Insight Communication	0.2410	0.1230	0.1420	0.2410	0.1230	0.1420
Style Imitation	0.1730	0.0150	0.1730	0.0140	0.1420	0.0012
Perception	0.0012	0.1420	0.0012	0.1230	0.1420	0.0012
Classification	0.0012	0.0450	0.0012	0.0012	0.0150	0.1420
Regression	0.1420	0.0012	0.1420	0.0140	0.1420	0.0120
Reinforcement	0.0120	0.0140	0.0120	0.1230	0.1420	0.2410
Semi-Supervised	0.2410	0.1230	0.2410	0.0150	0.1730	0.0150

**Table 11:** Final ranking of alternatives.

S. No.	Visualization Applications	Closeness Coefficients
1	Tableau	0.5425
2	Dundas BI	0.4856
3	Jupyter	0.3861
4	Zoho Reports	0.4163
5	Google Charts	0.4474
6	Visual.ly	0.5436

**Table 12:** Comparisons with other techniques.

S. No.	Visualization Tools	Fuzzy AHP-TOPSIS	Fuzzy AHP-VIKOR	Fuzzy AHP-ELECTRE
1	Tableau	0.5425	0.5356	0.5416
2	Dundas BI	0.4856	0.4789	0.4119
3	Jupyter	0.3861	0.3765	0.4065
4	Zoho Reports	0.4163	0.4265	0.4235
5	Google Charts	0.4474	0.4368	0.3168
6	Visual.ly	0.5436	0.5369	0.5269

## Data Availability

On reasonable request, the corresponding author will provide the information supporting the research study’s conclusions.
